# The role of community-based health insurance on healthcare seeking behavior of households in Addis Ababa, Ethiopia

**DOI:** 10.1016/j.pmedr.2023.102234

**Published:** 2023-05-15

**Authors:** Genanew Kassie Getahun, Kumlachew Kinfe, Zewdu Minwuyelet

**Affiliations:** Kotebe Metropolitan University, Menelik II Medical and Health Science College, Addis Ababa, Ethiopia

**Keywords:** Community based health insurance, Healthcare seeking behavior, Associated factors, Addis Ababa, Ethiopia

## Abstract

Community-based health insurance is a new and promising concept for delivering easily accessible, affordable, and efficient healthcare services. More importantly, a significant risk pooling arrangement between the wealthy and poor. So far, little has been done regarding community-based health insurance and healthcare seeking behavior in developing countries. Therefore, the aim of this study was to assess healthcare seeking behavior of households due to the introduction of the scheme and associated factors in Addis Ababa. Community-based cross-sectional study was conducted among 270 male and 207 female respondents. The data was collected using standardized and pretested questionnaire and it was analyzed using SPSS software. A bivariate and multivariable logistic regression analysis was applied to determine the relationship between healthcare seeking behavior and determinant variables. Finally, statistical association was declared with a p-value of less than 0.05 in the multivariable logistic regression analysis. The proportion of people who had appropriate healthcare-seeking behavior was 47.31% (95 %CI: 43.27–51.39%). Furthermore, having more than four children [AOR: 0.171, 95%, CI:(0.403–0.99)], being Muslim [AOR: 1.712, 95 %CI: (1.117 2.625)], owning a government house [AOR: 4.472, 95%, CI: (2.037–9.819)], and having under-five children [AOR: 0.548, 95%, CI: (0.375–0.801)] were factors significantly associated with healthcare-seeking behavior. Even though this study was conducted in an urban area, it showed that the magnitude of appropriate healthcare-seeking behavior was low. Therefore, the government of Ethiopia should work to improve housing conditions and family planning provision for the residents to improve the healthcare seeking behavior of community-based health insurance users.

## Highlights

• Community-based health insurance scheme is a strategy that provides financial protection against health-related poverty.
• Little has been known about the role of community-based health insurance on healthcare seeking behavior in Ethiopia.
• According to the findings of this study, the proportion of people who sought appropriate healthcare was low (47.3%).

## 1 Introduction

Health insurance has been advocated worldwide to increase access to healthcare services since the 1990s because it prevents patients from paying premiums directly and spreads the financial burden among all the insured (Tesfay, 2014). The community-based health insurance (CBHI) scheme is an emerging strategy for providing financial protection against health related poverty (Kebede & Geberetsadik, 2019). It is a healthcare funding option that may help to broaden coverage and improve health-care access in rural and underserved areas (Bennett, 2004).

CBHI (Community Based Health Insurance) is a novel idea for offering financial protection against the costs of disease and improving access to high-quality health care for low-income households and the informal sector that are not protected by formal insurance (Donfouet & Mahieu, 2012). As a strategy for achieving universal health coverage, some African countries have recently introduced health insurance to improve citizens' access to health care, mobilize resources for service improvement, and ultimately contribute to improved health quality (Wiesmann & Consultant, 2014). Ghana, Senegal, and Rwanda are a few of the African countries that have tried out CBHI as a national health program. Rwanda is one of the few African countries that has gone to great lengths to introduce CBHI (Shimeles, 2010).

Ethiopia has begun establishing a comprehensive and sustainable risk protection system with healthcare financing mechanisms tailored to the country's needs as financial risk protection is a critical component of Universal Health Coverage (UHC) in 2011 (Shigute et al., 2020). Following the implementation of CBHI, evidence generally indicated a positive effect and direct relationship on healthcare seeking and utilization rates (Studies, 2014). For the success of CBHI, continuous research work can help the concept improve and flourish in the future (Roy & Sarkar, 2018).

Enrollment of CBHI has reached 45.5% of the target households for the program in less than two years. CBHI's uptake was enhanced by connecting it to current social services and government systems (Taddesse et al., 2020). CBHI-related factors and their effect on the healthcare system altered household access to healthcare services, improved healthcare seeking behavior, and improved the quality of service provision (H, 2007, Jembere, 2018).

Some evidence suggests that both SHI and CBHI have a positive impact on healthcare quality, social inclusion, and reduce health-care costs. It may also increase routine and preventive care in general via reducing financial burdens by allowing them to rely on insurance coverage rather than spending a large portion of their income on healthcare (Al-Hanawi et al., 2020, Chomi et al., 2014). According to a study conducted in west Ethiopia, households that were members of a risk-sharing institution used health services at a higher rate, which directly increased their healthcare seeking behavior (Atnafu et al., 2018, Spaan et al., 2012). According to Molla Yismaw et al., the scheme encouraged rural households to seek healthcare, particularly for excluded people, such as the poor and chronically ill. Thus, the poor can benefit from indigent entitlement, and the chronically ill can be cross-subsidized by the healthy (Jembere, 2018).

As a result, the CBHI scheme has become a vehicle for achieving universal health coverage by avoiding out-of-pocket expenses and increasing healthcare seeking behavior (Kamau & Njiru, 2014). Public funding to subsidize premiums for the poor; promoting increased revenue collection from the “healthy and wealthy” to enhance cross subsidization and risk pooling; improving CBHI management; and improving the quality of care and health seeking behavior of households (mabrate et al.., 2015, Osei Asibey and Agyemang, 2017, Sohn and Jung, 2016). Besides, health care utilization is expected to be higher among CBHI user households. However, there is no a research work that indicates the CBHI users’ health-care seeking behavior in Ethiopian including Addis Ababa. As a result, this study can serve as a starting point for more in-depth study on the subject. Therefore, the aim of this study was to assess the healthcare seeking behavior of households in Addis Ababa, Ethiopia in 2022, following the introduction of the community-based health insurance scheme.

## 2 Methods

### 2.1 Study area and population

The study was conducted in Addis Ababa, Ethiopia. Addis Ababa is one of the most populous cities in Africa. It is situated at 38.763611 '' E longitude, 9° 0' 19.443 9.005401'' N latitude. The city is composed of 11 sub-cities with an estimated population of 5,006,000. Among these, 47.5% were males and the remaining 52.5% were females (Ethiopian Public Health Institute (EPHI) & ICF, 2021). According to the Addis Ababa city CBHI scheme office report, there were an estimated 897,750 head households in the city and 226,559 households were covered by community-based health insurance schemes in 2021 (Health & Improvement, 2021). The city had a lower rate of infant mortality than the national average, and over 98% of homes in the city had access to clean drinking water (CSA, 2012). The actual data collection was carried out from July 5^th^ to August 30^th^, 2022 using a community-based cross-sectional study design among household heads in the selected sub-cities of Addis Ababa, Ethiopia as a study population.

## 3 Eligibility criteria

The study included people over the age of 18 and heads of household enrolled in CBHI, and who had lived in Addis Ababa for at least 6 months prior to the survey. However, the study excluded household heads who used the fee waiver service and membership periods of less than 6 months, and people who were seriously ill and unable to communicate.

## 4 Sample size determination and sampling procedure

Sample size was calculated using a single population proportion formula. Since no research has been done to show a relationship between CBHI and healthcare seeking behavior in the Addis Ababa context, a 50% proportion was used to obtain a bigger sample size with a 95% confidence level and a 5% margin of error.

$$n=\frac{{\left(Z\frac{\alpha }{2}\right)}^{2}P\left(1-P\right)}{d^{2}}$$

Finally, the minimum sample size was 634 household heads, adding a 10% non-response rate and a design effect of 1.5.

A three-stage random sampling technique was employed to select the study participants. A simple random sample technique was applied at each stage to eliminate selection bias. Three sub-cities were selected and considered as a primary sampling unit. Then, each district in the selected sub-city was assumed as a secondary sampling unit, and households were the third sampling unit. By the lottery method, three sub-cities and twelve districts were selected. Based on the CBHI registration book, heads of households were selected by simple random sampling. The CBHI registration book was used as a sampling frame. Then proportional allocation was done for each selected district.

### 4.1 Study variables and definition

#### 4.1.1 Dependent variable

Healthcare seeking behavior of CBHI users

#### 4.1.2 Independent variables

**Predisposing (socio-demographic) factors:** like age, sex, marital status, past illness, education, household size, occupation

**Enabling factors:** like income, access to health care, location, price of services.

Perceived ill health and morbidity-related factors: Formal treatment of public health facilities or private health facilities

**Community-based health insurance:** is a scheme in which community members prepay for healthcare services, entitled to own the scheme, and control its management. It focuses on solidarity and mutual collective pooling of resources to share the financial costs of healthcare services.

**Healthcare-seeking behavior:** is a series of decisions about seeking (using) healthcare that are influenced by a variety of factors. It can be appropriate if the service is obtained from any healthcare facility and inappropriate if the service is from self-treatment, a traditional healer, or someone who did not know where to go (Fikre Bojola et al., 2018).

### 4.2 Data quality assurance

Data was collected using structured questionnaire, which was adopted from different literature with slight modification (Fikre Bojola et al., 2018, Jembere, 2018, Shimeles, 2010). The questionnaire consisted of five sections: socio-demographics, knowledge, attitudes, healthcare seeking, and overall satisfaction. Data was collected through face-to-face interviews after providing information about the purpose and confidentiality. Data quality was ensured through training of data collectors, supervisors, and pre-testing of questionnaires. A pretest was conducted among 5% of the sample in Yeka sub city district nine that was not included in the actual data and modification was done based on the pretest finding before the beginning of the actual data collection. Close supervision of data collectors was done and data was checked for completeness by the principal investigator.

### 4.3 Data entry and analysis

Data was cleaned, coded and entered into Epi-Data version 3.1, then exported to SPSS for analysis. Frequencies, proportions, and summary statistics were used to describe the study populations. A bivariate logistic regression analysis with a 95% confidence interval was performed to identify potential candidate variables using the crude odds ratio. In bivariate analysis, a p-value of less than 0.25 was used as a cut-off point to select candidate variables for the final model multivariable logistic regression. The normality of data was tested using Hosmer Lemeshow goodness of fit test. Finally, statistical significance was declared with a p-value of less than 0.05 as the cut-off point.

### 4.4 Ethical consideration

The ethical principles outlined in the Declaration of Helsinki guide the entire research process, which states that “it is the duty of the physician to promote and safeguard the health, well-being, and rights of patients, including those who are involved in medical research” (Holstila et al., 2016). The researchers secured ethical approval from Kotebe Metropolitan University, Menelik II Medical and Health Science College research and ethical review board with reference number KMU/18/10/3001. Official letters were obtained from the Addis Ababa health bureau. Data was collected after having informed written consent from each respondent.

## 5 Results

### 5.1 Scio-demographic characteristics of respondents

Overall, 577 respondents participated in this study, yielding a response rate of 91%. Male heads of households accounted for 64.1% of the total, while female heads of households accounted for 35.9%. The majority of respondents (45.9%) were over the age of 46 years, followed by the age categories of 36–45 years and 26–35 years, which accounted for 20.3 percent and 19.2 percent, respectively. Age groups were formed based on the assumption of youth, young adults, adults, and old age groups. In terms of marital status, 365 (63.3%) of the respondents were married, while 13% were never married, and the remaining 68 (11.8%) and 69 (12%) respondents were widowed or divorced, respectively (Table 1).

**Table 1:** Socio-demographic characteristics of respondents.

Variables	Category	Frequency	Percent
Sex	Male	370	64.1
	Female	207	35.9
Age (in years)	18–25	27	4.7
	26–35	119	20.6
	36–45	166	28.8
	46+	265	45.9
Religion	Orthodox	291	50.4
	Muslim	166	28.8
	Catholic	36	6.2
	Protestant	84	14.6
Marital status	Single	75	13.0
	Married	365	63.3
	Divorced	69	12.0
	Widowed	68	11.8
Family size	<4 members	455	78.9
	>4 members	122	21.1
Educational level	Illiterate	121	21.0
	read and write	49	8.5
	primary education	118	20.5
	secondary education	199	34.5
	diploma and above	90	15.6
Source of income	Farming	82	14.2
	commercial activity	204	35.4
	daily laborer	106	18.3
	Other(specify)	185	32.1
Ownership of housing	Private	249	43.1
	Rent	188	32.6
	Government	114	19.8
	Others(specify)	26	4.5
Monthly income (ETB)	<1170.00	46	8.0
	>1170.00	531	92.0

### 5.2 Motivation and reasons for CBHI membership

Seven questions were asked of respondents to determine why they joined the CBHI scheme. Multiple choices were given to identify various reasons why they became members of the CBHI scheme, with the two most common reasons being: first, 59.1% joined the scheme because premium payments were lower than out of pocket payments; and second, 42.6% joined the scheme because illness and injuries occur frequently in the household. Respondents were asked if any members of the household had been sick in the previous six months, and a significant number of respondents (96%) reported illness or injuries within the household. In addition, when asked whether they sought therapy or not, 89.4% reported that they sought treatment. In this study, families that encountered illness or injuries for family members were asked what their first treatment responses were for the occurrences of disease or injuries, and the majority of households (49.5%) reported that they have visited contemporary health-care facilities (Table 2).

**Table 2:** Motivation of households due to the introduction of CBHI scheme.

Do CBHI scheme motivate you?	Frequency	Percent
Yes	363	62.9
No	214	37.1
**Reason for your motivation (363)**		
Exemption of payment at the time of service	192 53	
Quality of health care improved	156 43	
Others (specify)	15 4	
**What was the reason for your demotivation? (2 1 4)**		
limited health service availability and drug supply	86 40	
long waiting time to get services	81 38	
low availability of diagnostic and lab service	41 19	
mistreatment by health providers for members	6 3	

### 5.3 The effect of CBHI scheme on healthcare seeking behavior

Healthcare seeking behavior is more prominent in the process of using a formal (modern) healthcare facility. The CBHI plan has the ability to promote immediate health-care use by modern health-care institutions. Respondents were asked whether the CBHI program allows people to obtain modern healthcare treatments on a regular basis to support the aforesaid rationale. The vast majority of them (62.9%) revealed that the CBHI scheme encourages people to seek healthcare from contemporary health facilities. Furthermore, respondents who believed CBHI motivated households to seek modern health services were asked why they were motivated to seek modern healthcare on a regular basis, and the majority (53%) of respondents believed that lack of payment was a major factor (Table 3).

**Table 3:** Health seeking behavior of CBHI users.

Have you or members ill in the past six months?	Category		Frequency	Percent
	Yes		554	96.0
	No		23	4.0
**Did your family members seek treatment?**	Yes		516	89.4
	No		61	10.5
**What was/were your family member/immediate treatment?**	visit modern health care facility	Yes	516	89.4
		No	44	7.6
	visit traditional healer	Yes	350	60.2
		No	227	39.8
	use home healing	Yes	145	25.0
		No	432	75.0
	go to holy water	Yes	31	5.4
		No	546	94.6
**Do you believe medication should be finished?**		Yes	459	79.5
		No	118	20.5

### 5.4 Effect of the CBHI scheme on treatment choices

Respondents were asked to identify what kind of treatment actions they undertake when feeling ill before and after becoming a member of the scheme to examine the choice of treatment households employed. A significant number of respondents (78.8%) reported that they used home healing before becoming a CBHI member. On the other hand, nearly half of the respondents (56%) reported that they used home healing after becoming a CBHI member. After families join the CBHI program, modern healthcare facilities take the lion's share of visits to healthcare institutions for disease and injury treatment. The reason behind such action is the free accessibility of modern healthcare at the time of services. Nearly all respondents considered visiting modern healthcare institutions as their first and best option for treating illness after they became members of CBHI (Table 4).

**Table 4:** Household treatment habits before and after CBHI membership.

**Before CBHI which type of treatment your family members employed?**	Visit traditional healer		**Frequency**	**Percent**
		Yes	27	4.7
		No	550	95.3
	Visit modern health care facility	Yes	50	8.7
		No	527	91.3
	Use home healing	Yes	540	5.4
		No	37	94.6
	Go to holy water	Yes	37	6.4
		No	540	93.6
	Forgone minor illness or injury	Yes	31	5.4
		No	546	94.6
**Which type of treatment your family use after CBHI?**	Visit traditional healer	Yes	40	6.9
		No	537	93.1
	Visit modern health care facility	Yes	398	69.0
		No	179	31.0
	Use home healing	Yes	222	38.5
		No	355	61.5
	Go to holy water	Yes	50	8.7
		No	527	91.3

### 5.5 Level of healthcare-seeking behavior

**Fig. 1:**
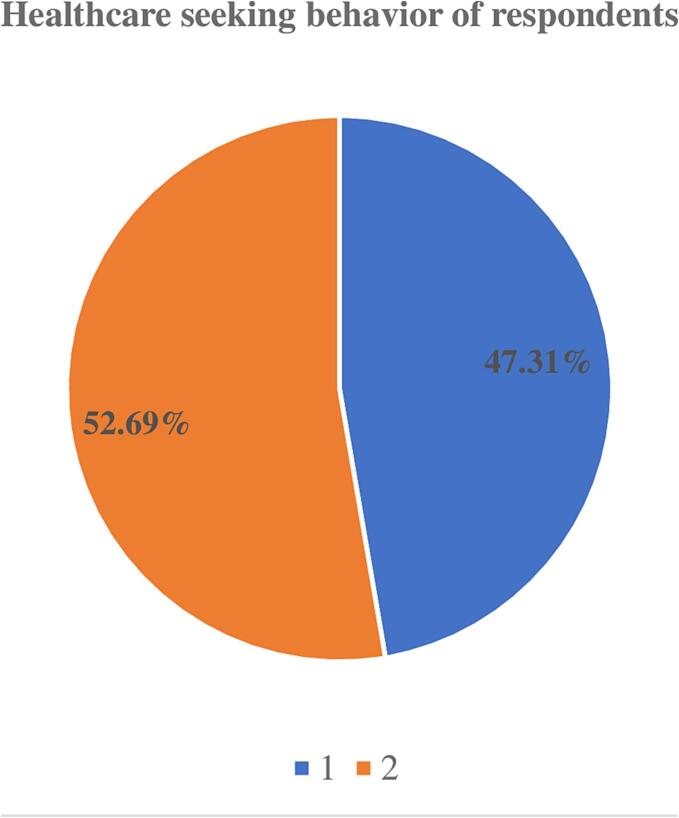
Healthcare seeking behavior of CBHI users in Addis Ababa, Ethiopia.

Out of the total respondents, 273, 47.31% (95% CI: 43.27–51.39%) had appropriate healthcare seeking behavior. On the other hand, 304 (52.69%) had inappropriate health seeking behavior (Fig. 1).

### 5.6 CBHI knowledge, attitude, and satisfaction of study participants

**Fig. 2:**
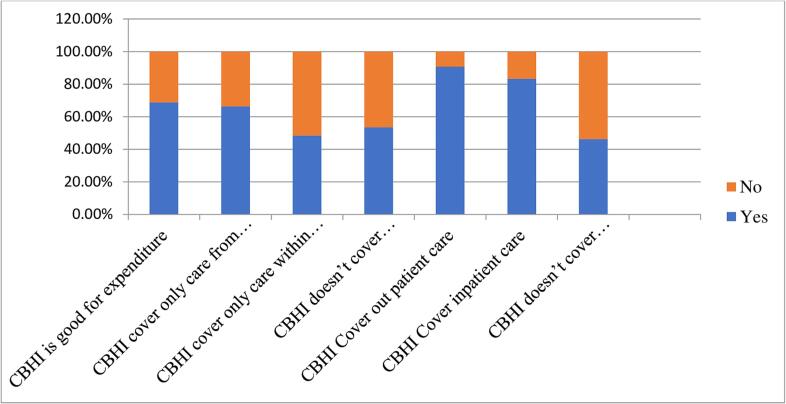
Knowledge of CBHI users about benefit packages in Addis Ababa, Ethiopia.

Of the total respondents, 63.6% had good knowledge, implying that respondents knew benefit packages of four and above. On the other hand, 36.4% had poor knowledge (Fig. 2). Furthermore, of the total respondents, 79.9% reported a favorable attitude towards CBHI, and 53.6% of the study participants reported being satisfied with the CBHI scheme. According to the findings of the study, members' satisfaction with health facilities has increased since the introduction of CBHI. Only 41.6 percent of members were satisfied with the services they received prior to the CBHI scheme. However, following the implementation of CBHI, this percentage increased to 63.8%, owing primarily to a lack of payment and good health care treatment. However, some households (22.3%) were dissatisfied due to a lack of drug supply and additional out-of-pocket expenses.

### 5.7 Factors associated with healthcare seeking behavior among CBHI users

Factors associated with healthcare seeking behavior were identified using binary logistic regression analysis at a p-value < 0.25. As a result, sex of respondents, religion, marital status, participation in CBHI related meetings, family size, ownership housing, knowledge, attitude, satisfaction, a desire of CBHI users to cut off CBHI membership, the presence of chronic illness in the family, and the availability of under five children in the family were significantly associated and selected for multivariable logistic regression.

Finally, five independent variables have a significant relationship with appropriate healthcare seeking behavior, namely: religious affiliation, family size, housing condition, and the presence of under-five children, were significantly associated with healthcare seeking behavior of CBHI users at a p-value of < 0.05 (Table 5).

**Table 5:** Factors associated with healthcare seeking behavior of respondents.

Variables	Category	HCSB		COR (95%, CI)	AOR (95%, CI)
		Inappropriate	Appropriate		
Sex	Male	183	187	1	1
	Female	121	86	0.696(0.493,0.981)	0.718(0.496,1.039)
Religion	Orthodox	171	120	1	1
	Muslim	80	86	1.53(1.044,2.248)	1.712(1.117,2.62**
	Catholic	12	24	2.85(1.37,5.92)	2.89(1.362,6.142) *
	Protestant	41	42	1.46(0.895,2.382)	1.341(0.791,2.274)
Marital status	Single	36	39	1	1
	Married	207	158	0.705(0.428,1.159)	0.639(0.377,1.085)
	Divorced	35	34	0.897(0.466,1.725)	0.700(0.347,1.411)
	Widowed	26	42	1.49(0.766,2.904)	1.278(0.625,2.613)
Family size	<4	233	222	1	1
	>4	71	51	0.754(0.503,1.129)	**0.634(0.403,0.999)**
Ownership of housing	Private	144	105	1	1
	Rent	106	82	2.083(1.035,4.189)	1.824(0.867,3.837)
	Government	41	73	4.794(2.281,10.07)	**4.472(2.037,9.81)**
	Others	13	13	2.692(0.992,7.305)	2.028(0.710,5.790)
Knowledge	Unfavorable	124	86	1	1
	Favorable	180	187	1.498(1.063,2.11)	1.336(0.881,2.025)
Attitude	Unfavorable attitude	51	65	1	1
	Favorable attitude	253	208	0.645(0.428,0.972)	0.899(0.534,1.516)
Satisfaction	Dissatisfied	126	142	1	1
	Satisfied	178	131	0.653(0.470,0.9080)	0.830(0.538,1.281)
Did you participate in CBHI related meeting?	Yes	91	85	0.945(0.663,1.37)	1.052(0.694,1.596)
	No	213	188	1	1
Do you plan to cut your CBHI membership?	Yes	97	116	0.634(0.451,0.891)	1.252(0.788,1.988)
	No	207	157	1	1
Is there any chronic illness in the family?	Yes	124	125	0.816(0.586,1.135)	0.990(0.680,1.442)
	No	180	148	1	1
Are there under five children in the family?	Yes	158	88	2.275(1.621,3.194)	0.548(0.375,0.801)*
	No	146	185	1	1

**Note: HCSB:** is the dependent variable**, COR:** Crude odds ratio, **AOR:** Adjusted odds ratio, *: Significant result, **1:** Reference category, **: p-value <=0.01, *: p-value<=0.05.

## 6 Discussion

This study aimed to identify the relationship between healthcare seeking behavior and associated factors among households who were enrolled in community-based health insurance. Prior to joining CBHI, 8.7% of people used contemporary healthcare. However, after joining CBHI, this percentage jumped to 69%, which was consistent with research done in northern Ethiopia (Bhageerathy et al., 2017, Jembere, 2018). The possible reason could be that after paying the premium, members might be tempted to test the services that were promised.

This study's finding revealed that 47.31% of the study participants had appropriate healthcare-seeking behavior. This was comparable to that of research finding from northern Ethiopia (Tesfay, 2014). However, it was lower than a result found from the Tehuldere district of northern Ethiopia at 71.5% (Jembere, 2018), the Sidama zones of south west Ethiopia at 72.8% (Begashaw et al., 2016) and the Dale district southern Ethiopia at 80.7% (Fikre Bojola et al. 2018). The difference in healthcare seeking behavior might be due to the impact of COVID-19, which created fear in the community of seeking treatment from modern health facilities. Besides, the difference in study period and the sociodemographic characteristics might be responsible for the discrepancy. For instance, in the study's Tehuldere district, most of the study participants were males, and in Ethiopian culture, men have more decision-making power for treatment and public actions (processes during membership).

Based on the result of this study household heads with a family size greater than four members had a 63% higher risk of inappropriate healthcare seeking behavior than those with a family size of less than four members. It was in line with a finding from a population-based survey conducted in North West Ethiopia (Atnafu et al., 2018). This might be due to the fact that those who have larger family members might give less attention to members of the household in terms of their nutritional needs, and this makes them prone to illness and an increased probability of using inappropriate medical care. This finding is in support of research done in Ghana and Kenya (Muriithi, 2013, Osei Asibey and Agyemang, 2017).

In addition, housing condition showed a significant association with appropriate healthcare seeking behavior where households having a government house had more than four times the odds of having appropriate healthcare seeking behavior when compared with households living in a rental house, which is consistent with a study finding from Singapore and Mumbai (Bardhan et al., 2018, Chan et al., 2018). The possible reason for this could be that households with their own house might spend more money on their health needs than households that spend money on house rent might invest the least in their health needs.

On the other hand, households that had under-five children showed a significant association with healthcare seeking behavior. Hence, households that had under five children were more likely to have appropriate healthcare seeking behavior compared with their counterparts. It was in line with the study findings done in the Amhara region's Ensaro district (Dagnew et al., 2018, Sisay et al., 2015). The reason behind it could be due to the fact that households who have under-five children might give more emphasis to their children's health.

Moreover, being a Muslim household head had nearly two times the odds of appropriate healthcare seeking behavior compared with orthodox Christian followers. This finding was supported by a study finding done in India's urban health centers (Patil et al., 2016, Vu et al., 2016). It is possible that orthodox religious followers might use additional alternatives, such as holy water.

**Limitation:** This study relied on the six-month experience of health care needs of the respondent prior to the study, making it vulnerable to recall bias. For the sickness report, the study used an individual's viewpoint and an oral report. The study design was not robust enough to distinguish between the cause and impact relationship.

## 7 Conclusion

The result of this study showed that the proportion of respondents who had appropriate healthcare seeking behavior among CBHI users was low. Moreover, family size, the presence of under-five children, religious affiliation, and housing ownership was significantly associated with healthcare seeking behavior. Therefore, the government of Ethiopia should work hard to improve housing conditions and contraceptive provisions for residents to improve the healthcare seeking behavior of CBHI users.

## 8 Consent for publication

Not applicable.

## 9 Data availability

The datasets used to support the findings of this study are attached with the manuscript.

## Funding

The study has no funding source.

## CRediT authorship contribution statement

**Genanew Kassie Getahun:** Conceptualization, Data curation, Visualization, Investigation, Writing – original draft, Writing – review & editing. **Kumlachew Kinfe:** Conceptualization, Supervision, Data curation, Writing - original draft, Writing - review & editing. **Zewdu Minwuyelet:** Methodology, Supervision, Writing – review & editing.

## Declaration of Competing Interest

The authors declare that they have no known competing financial interests or personal relationships that could have appeared to influence the work reported in this paper.

## Data Availability

Data will be made available on request.
